# Topical Corticosteroids a Viable Solution for Oral Graft versus Host Disease? A Systematic Insight on Randomized Clinical Trials

**DOI:** 10.3390/medicina56070349

**Published:** 2020-07-14

**Authors:** Arin Sava, Andra Piciu, Sergiu Pasca, Alexandru Mester, Ciprian Tomuleasa

**Affiliations:** 1 Department of Oral Rehabilitation, University of Medicine and Pharmacy “Iuliu Hatieganu”, 400012 Cluj-Napoca, Romania; arin_sava@yahoo.com; 2 Department of Medical Oncology, University of Medicine and Pharmacy “Iuliu Hatieganu”, 400012 Cluj-Napoca, Romania; piciuandra@gmail.com; 3 Department of Hematology, University of Medicine and Pharmacy “Iuliu Hatieganu”, 400012 Cluj-Napoca, Romania; pasca.sergiu123@gmail.com (S.P.); ciprian.tomuleasa@gmail.com (C.T.); 4 Department of Oral Health, University of Medicine and Pharmacy “Iuliu Hatieganu”, 400012 Cluj-Napoca, Romania

**Keywords:** oral graft versus host disease, topical corticosteroids, dexamethasone, clobetasol, budesonide

## Abstract

*Background and Objectives:* This research attempts to provide a clear view of the literature on randomized clinical trials (RCTs) concerning the efficacy of topical dexamethasone, clobetasol and budesonide in oral graft versus host disease (GVHD). *Materials and Methods:* An electronic search of the PubMed, Web of Science and Scopus databases was carried out for eligible RCTs. Studies were included if they had adult patients with oral GVHD treatment with topical corticosteroids, and if the RCT study was published in English. The Cochrane Risk of Bias tool was used to assess the quality of these studies. Overall, three RCTs were included (an Open, Randomized, Multicenter Trial; a Randomized Double-Blind Clinical Trial; and an Open-Label Phase II Randomized Trial). *Results:* The trials involved 76 patients, of which 44 patients received topical dexamethasone, 14 patients received topical clobetasol and 18 patients received topical budesonide. Topical agents were most frequently used when oral tissues were the sole site of involvement. It appears that the best overall response is present for budesonide with no difference between the four arms, followed by clobetasol, and then by dexamethasone. The limitation of the current study is mainly represented by the fact that overall response was derived in two of the studies from other parameters. Moreover, both budesonide and clobetasol were used in only one study each, while two assessed dexamethasone. *Conclusions:* Based on the clinical trials, all three agents seem to be effective in treating oral GVHD and had a satisfactory safety profile. There is still a need for assessing high quality RCTs to assess the efficacy of these therapies on a larger cohort.

## 1. Introduction

Allogeneic hematopoietic cell transplantation (allo-HCT) is protocol treatment for hematological cancers and also for non-malignant disorders [1]. Graft versus host disease (GVHD), which is an important complication of allo-HCT is induced by the interactions of the host’s immune system and transplanted donor cells. According to the time of appearance and clinical signs, GVHD is classified as acute or chronic [2,3]. Chronic GVHD (cGVHD), most frequently occurs ≥100 days after the transplant, affecting 25–40% of long-term HCT survivors [4]. cGVHD is classified as limited when single organs are involved, such as the skin or liver, and extensive when multiple affection occurs, in organs such as the skin, liver, eyes, salivary glands, oral mucosa and others [5,6,7].

Oral manifestations occur in about 80% of patients with extensive cGVHD. Most frequent oral lesions are erythema, atrophy of the mucosa, oral lichen planus, oral mucositis, xerostomia and oral infections [6,8,9]. Oral mucositis is an inflammatory reaction of the oral mucosa, often occurring after high doses of chemotherapy, radiation therapy and/or stem cell transplantation. In hematopoietic cell transplantation (HCT) patients, mucositis may often occur along the entire orodigestive tract. The prevalence of oral mucositis is stated to be at 30–70% after chemotherapy, up to 90% after HCT [10]. Oral lichen planus aspects may vary from white lacey patches to open sores, involving the tongue and inner surface of cheeks [10,11].

Oral involvement could represent the only manifestation of the cGVHD or could be comprised in a multitude of chronic symptoms. cGVHD may appear on the oral mucosa (e.g., oral verruciform xanthoma, erytroplakia), at the salivary glands (e.g., multiple mucoceles on the soft palate, hyposalivation, xerostomia, impairment in the quality of saliva, gland swelling) and in musculoskeletal apparatus disfunction [11,12,13].

Dysphagia is one of the most debilitating symptoms and is induced by the pain associated with oral mucositis [9]. Local palliation of the oral symptoms is achieved either by systemic therapy, or topical treatment, or both. In this light, the use of topical agents determines the reduction of the oral symptoms, determines the reduction of the systemic immunosuppressant doses, minimizes their side effects and increases the healing process [2,10].

Although topical corticosteroids are not specifically approved for treatment of oral cGVHD, they are used to treat these symptoms, based on previous experiences that prove their well accepted use in other mucosal conditions [13,14,15].

Given the availability of limited data, the present research attempts to provide a systematic approach of literature including randomized clinical trials (RCTs) concerning the efficacy of topical dexamethasone, clobetasol and budesonide in oral GVHD.

## 2. Materials and Methods

We conducted a systematic review using RCTs to compare topical dexamethasone, clobetasol and budesonide for oral GVHD. This study was in accordance with the PRISMA (Preferred Reporting Items for Systematic Reviews and Meta-Analyses) guidelines and the Cochrane Collaboration format [16,17].

Three databases (PubMed, Web of Science, Scopus) were searched to identify eligible articles from inception to February 2020, using keywords (“dexamethasone”, “clobetasol”, “budesonide”, “topical corticosteroids”, “corticosteroids”, “oral graft versus host disease”, “oral GVHD”) combined with a Boolean term (“AND”) as follows: “dexamethasone AND oral graft versus host disease”; “dexamethasone AND oral GVHD”; “clobetasol AND oral graft versus host disease”; “clobetasol AND oral GVHD”; “budesonide AND oral graft versus host disease”; “budesonide AND oral GVHD”; “topical corticosteroids AND oral graft versus host disease”; “topical corticosteroids AND oral GVHD”; “corticosteroids AND oral graft versus host disease”; and “corticosteroids AND oral GVHD”. Articles were evaluated by their titles and abstracts. The contents of the articles were assessed in order to determine if the studies met the inclusion/exclusion criteria. The full texts of the potentially relevant studies were retrieved and assessed. The reference lists of the chosen articles were manually searched to identify any other relevant studies that have been missed out using the search strategy.

The inclusion criteria used in the article selection were adult (≥18 years) oral GVHD; treatment with topical corticosteroids (dexamethasone, clobetasol, budesonide); RCT; and human studies, published in English. All other articles that did not complete the upper criteria were excluded from our research.

Two independent reviewers assessed the articles for eligibility and extracted the data using a standardized data extraction form. All lack of concordance was solved by a third reviewer. The following data were taken out: author, year, country, study type, sample size, mean age, male: female ratio, oral GVHD at baseline, treatment design, clinical response, side effects, outcome.

The Cochrane Collaboration’s “Risk of Bias” tool 2.0 was used to assess the quality of these studies [17]. For every RCT included, a risk of bias was provided for the following domains: random sequence generation, allocation concealment, blinding of outcome assessment, incomplete outcome data, selective reporting and other bias. These domains were judged by two reviewers and were evaluated as low, unclear or high, and a third reviewer was invited to solve all unclear results.

## 3. Results

**Figure 1:**
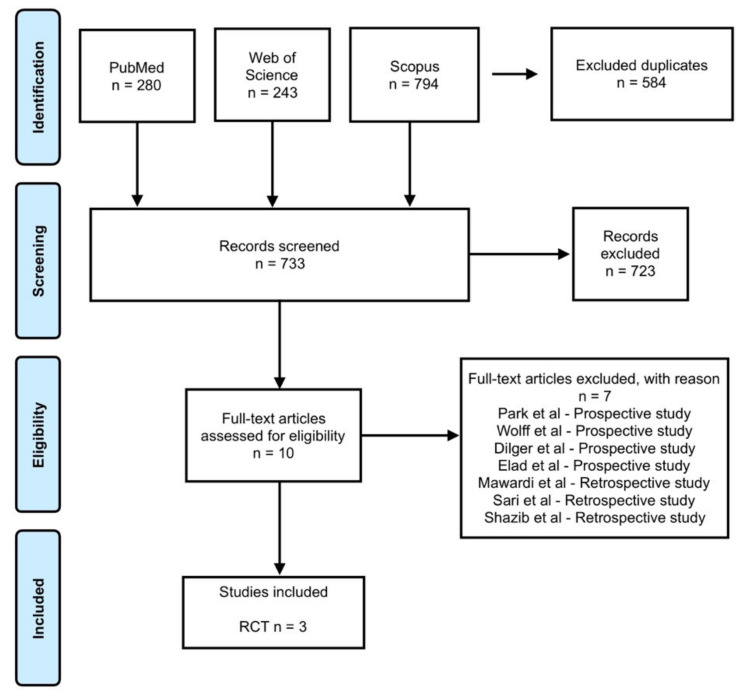
Flow chart of the study selection. RCT - randomized clinical trial.

The search strategy generated 1317 articles (Figure 1). After the exclusion of 584 articles, 733 articles were identified as eligible records. However, 723 articles were excluded because they did not fulfil all eligibility criteria. Therefore, 10 articles resulted as eligible, but 7 were excluded because they were prospective [18,19,20,21] or retrospective [22,23,24] studies. In the end, three RCTs were included [25,26,27].

The three RCTs included were an Open, Randomized, Multicentre Trial [25], a Randomized Double-Blind Clinical Trial [26] and an Open-Label Phase II Randomized Trial [27]. They were published between 2012 and 2016, involving a total of 76 patients, of which 44 patients received topical dexamethasone, 14 patients received topical clobetasol and 18 patients received topical budesonide. The studies were conducted in Israel/Germany [25], Brazil [26] and the USA [27]. The mean age of the participants varied from 43.8 to 55 years and the sex ratio was female dominant.

Oral GVHD diagnosis was done on different parameters across the included studies: WHO toxicity oral/gastrointestinal, modified oral mucosal rating scale (mOMRS), Oral Mucositis Assessment Scale (OMAS), National Institute of Health (NIH) oral cavity severity score, mucosal score and oral symptoms score. Oral lesions involved in GVHD were erythema, atrophy, ulcer, lichen, hyperkeratosis, pseudomembrane, edema and mucocele, appearing as a mucus cyst on the soft palate, on the labial and buccal mucosa. Clinical response to these agents were 61% for WHO toxicity oral/gastrointestinal, 50–61% for mOMRS, 69% for OMAS and 50% for NIH oral cavity response. Side effects reported were cheilitis, esophagitis, fungal infections, taste alteration, burning sensations and oral cavity pain. Additional data can be found in Table 1.

**Figure 2:**
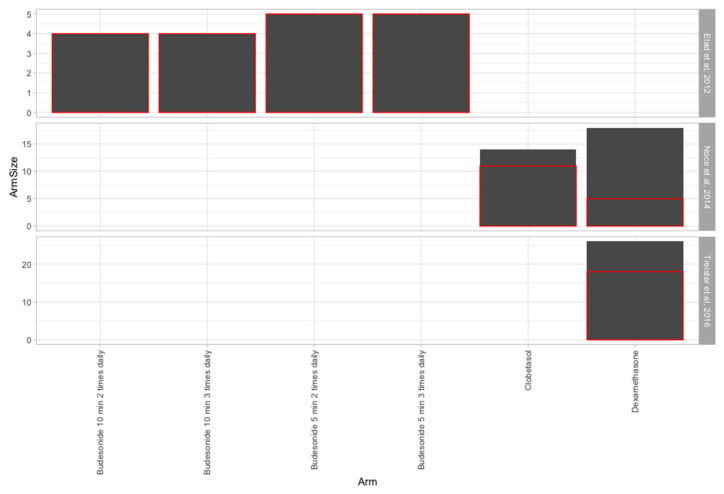
The overall response between the arms of the three studies included.

Figure 2 represents the overall response between the included studies. Red rectangles represent the proportion of patients that presented a response. Because of the heterogeneity of the studies, we considered for Elad et al. [25] the mOMRS any response, for Noce et al. [26] the symptomatic response and for Treister et al. [27] the overall response described by the authors. It appears that the best overall response is present for budesonide with no difference between the four arms, followed by clobetasol and then by dexamethasone. The limitation of the current study is mainly represented by the fact that the overall response was derived in two of the studies from other parameters. Moreover, both budesonide and clobetasol were used in only one study each, while two assessed dexamethasone.

**Figure 3:**
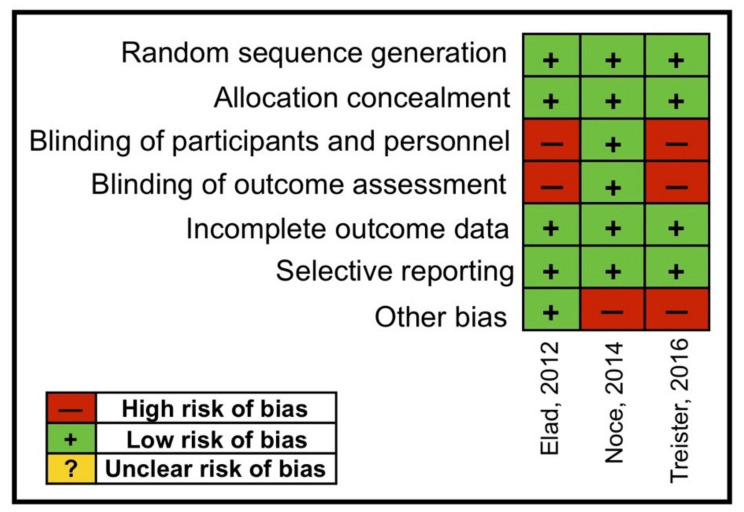
Risk of bias for the RCTs.

Overall, the three included RCTs were considered at “risk of bias” because of the lack of blinding of study participants, blinding of the outcome data and other bias. Figure 3 shows the analysis for the risk of bias for RCTs.

## 4. Discussion

Up to this moment, in the current literature, there are two systematic reviews trying to assess the benefits of using topical agents in oral GVHD [10,28]. Albuquerque et al. observed that there are a limited number of RCTs, and, therefore, the evidence sustaining the use of topical agents for the inflammatory lesions in oral GVHD is low [10]. The same authors have stated that there is still a need for quality RCTs to assess the efficacy of these agents in GVHD [10]. The paper of Elsaadany et al. reported moderate evidence for the efficacy of topical agents for oral GVHD, showing minimal side effects of clobetasol followed by budesonide [28]. Our systematic review had a homogenous selection of randomized clinical trials allowing a calculation of the Cochrane risk of bias and of the overall response, and, therefore, giving a clear recommendation for a better efficacy of budesonide, compared to clobetasol and then dexamethasone.

Another powerful parameter we have evaluated from the RCTs included in our review is the safety profile of the therapy. In the study of Elad et al. [25], the use of topical budesonide (3 mg/10 mL) showed that this corticosteroid had a satisfactory safety profile. Topical budesonide mouthwashes improved the oral condition when it was applied for 5–10 min, 2/3 times per day. Regarding the response in all treatment arms, Elad mentioned that it was the same, in any length of exposure to treatment and in any frequency. Safety analysis was performed at a dosing schedule of budesonide 3 mg, three times per day, for a period of 10 min, representing the most important exposure to the drug [25].

In the study of Noce et al. [26], a significant improvement in the symptoms appeared comparing to the baseline after the use of corticosteroids, but with a significantly greater response in the topical clobetasol group compared to dexamethasone. The authors stated that the limitations of their research were the low number of subjects and other confounding variables. They indicate that these variables in further studies should be taken into account, with a larger sample size and stratification of subjects [26].

Treister et al. [27] observed in their study that the patients with dexamethasone obtained a response of 58%. The overall global response rates were reported to reach up to 81% including responses such as much better. In total, 96% of the patients reported the dexamethasone rinses as being well tolerated and the taste being “very pleasant” or “tolerable”. According to these results, the authors concluded that intensive topical therapy with this agent is efficient for managing oral chronic GVHD and should be used as a first line therapy [27].

Other topical therapies studied in the literature include triamcinolone, fluocinonide, betamethasone, tacrolimus and prednisolone, fluocinonide, halobetasol prepared as a gel or cream and topical platelet-rich gel, with various results. Because of their lack of availability in all countries we have used in our inclusion criteria only the studies mentioning the most common and used steroids: budesonide, clobetasol and dexamethasone [11].

Our research tried to do a systematic review and a meta-analysis. The second objective was not able to be fulfilled because of the heterogeneity of the articles included. The low number of RCTs included in this review represents a major limitation in concluding on the efficacy of topical corticosteroids in oral GVHD and establishing a therapy protocol. On the other hand, several weaknesses were observed within the studies included. First, a variation in assessing oral GVHD parameters before/after topical agents was observed. The adjustments of the oral GVHD parameters is required. Secondly, the sample sizes were too small; a larger sample size should be used in future studies. Third, all RCTs included only chronic GVHD patients, excluding the acute alarming manifestations. This research was made evaluating only three databases and included only articles written in English, leading to a possible exclusion of other important data.

## 5. Conclusions

The purpose of the oral GVHD treatment is to reduce symptoms, maintain or improve the quality of life and reduce complications. Topical corticosteroids are most frequently used when oral tissues are the sole site of involvement. Based on the clinical trials, all three agents seem to be effective in treating oral GVHD and have a satisfactory safety profile. More RCTs with larger cohorts are needed to assess the efficacy of this topical agents.

## Figures and Tables

**Table 1:** Characteristics of the included randomized clinical trials (RCTs).

Author/Year/Country	Study Type	Sample Size	Oral GVHD at Baseline	Treatment Design	Clinical Response	Side Effects	Outcome
Elad et al., 2012, Israel/Germany	Open, Randomized, Multicenter	n = 18	Oral cGVHD WHO toxicity gastrointestinal/oral—grade 1 (n = 2)—grade 2 (n = 13)—grade 3 (n = 3) mOMRS (median)—26 OMAS (median)—1.9	Budesonide 3 mg/10 mL mouth rinse, for 8 weeks Arm A: 3 × 10 min daily Arm B: 3 × 5 min daily Arm C: 2 × 10 min daily Arm D: 2 × 5 min daily	WHO toxicity gastrointestinal/oral—61% mOMRS—61% OMAS—69%	Cheilitis, esophagitis, fungal infection, taste alteration	Topical budesonide in oral cGVHD has a safety profile. Safety analysis supports a dosing schedule of 3 mg of budesonide 3 times a day applied for 10 min in the form of a mouthwash.
Noce et al., 2014, Brazil	Randomized Double-Blind Clinical Trial	Clobetasol group n = 14 Dexamethasone group n = 18	Oral lesions of cGVHD Erythema (n = 29) Atrophy (n = 26) Ulcer (n = 22) Lichen (n = 21) Hyperkeratosis (n = 19) Pseudomembrane (n = 3) Edema (n = 2) Mucocele (n = 14)	Clobetasol: patients rinsed their mouths with 5 mL of a solution of clobetasol propionate 0.05% administered with nystatin 100000 IU/mL for 28 days. Dexamethasone: patients rinsed with 5 mL of a solution of dexamethasone 0.1 mg/mL administered with nystatin 100000 IU/mL for 28 days.	In 53.9% of the cases, the use of clobetasol resulted in an improvement of at least 50% in the mOMRS total score. For dexamethasone, this result was observed in 26.7% of the patients.	Clobetasol: burning sensation Dexamethasone: burning sensation	Topical clobetasol or dexamethasone was efficacious in the reduction of symptoms related to oral cGVHD. Clobetasol was significantly more effective than dexamethasone in the symptomatic and morphologic improvement of oral lesions.
Treister et al., 2016, USA	Open-Label Phase II Randomized Trial	n = 26	Oral cGVHD NIH oral cavity severity score NIH oral mucosal score NIH oral symptom scores Oral biopsies	Dexamethasone was dispensed as a commercially prepared 0.5 mg/5 mL solution; 4 rinses per day for at least 28 days.	Overall response—69% Oral Mucosal Score Response—PR (8%), NR (88%), PD (4%) NIH oral cavity response—50%	Oral cavity pain	Topical dexamethasone is safe and effective at reducing the symptoms of oral cGVHD. Dexamethasone should at present be considered for first-line topical therapy in patients with previously untreated and symptomatic oral cGVHD.

HCT, hematopoietic cell transplantation; mOMRS, modified oral mucosal rating scale; NIH, National Institute of Health; OMAS, Oral Mucositis Assessment Scale; PR, partial response; NR, no response; PD, progressive disease.
